# Daily fluctuations in visual motion discriminability contribute to daily fluctuations in continuous visuomotor performance

**DOI:** 10.3389/fspor.2022.1009763

**Published:** 2022-11-02

**Authors:** Ayaka Takami, Ryoma Goya, Chisa Aoyama, Takaaki Komiyama, Toshitaka Kawajiri, Satoshi Shimegi

**Affiliations:** ^1^Graduate School of Frontier Biosciences, Osaka University, Osaka, Japan; ^2^Faculty of Sports and Health Science, Fukuoka University, Fukuoka, Japan; ^3^Graduate School of Medicine, Osaka University, Osaka, Japan; ^4^Center for Education in Liberal Arts and Sciences, Osaka University, Osaka, Japan

**Keywords:** daily fluctuations, visual motion discriminability, continuous visuomotor performance, visual information processing, ball sports

## Abstract

In ball sports such as table tennis, in which a ball moving at high speed is hit, an athlete's brain needs to process the motion information of the ball, predict the arrival point, and form a motor command to direct the racket there. Therefore, day-to-day fluctuations in visuomotor performance may be ascribed to fluctuations in visual motion discriminability, but it is not clear how the two are related. To examine this point, university table tennis players performed a motion direction discrimination (MDD) task and continuous visuomotor (CVM) task over 10 days as an estimation of visual motion discriminability and visuomotor performance, respectively. In the MDD task, using a joystick, participants distinguished the direction of a global coherent motion of target dots moving in the same direction on a PC monitor from innumerable dots moving in random directions. In the CVM task, participants hit sequential targets moving fast from right to left on the PC monitor by operating the cursor on the left side of the monitor up and down using the prehensile force of their thumb and index finger. The scores in the MDD and CVM tasks fluctuated day by day and showed a significant and moderate correlation between the MDD task score for the visual field in which the participants captured the target in the CVM task and the CVM task score. This correlation was confirmed even with the target moving from left to right. The fluctuations in the onset latency and the endpoint position of the cursor movement approaching the target were correlated with those of the visual motion discriminability, suggesting the contribution of motion vision to the speed and accuracy of the visuomotor performance. Moreover, these relationships were prominent in veteran players. For table tennis athletes, especially experienced players, fluctuations in the visual motion discrimination performance in a visual field specific for capturing a ball may be responsible for the fluctuations in continuous visuomotor (striking) performance.

## Introduction

It is well-known that the performance of athletes fluctuates day by day. However, the cause of these fluctuations is unknown, making it difficult to take countermeasures. One theory attributes the cause to daily fluctuations in physical (physiological) factors such as endurance (1, 2) and muscle strength (3, 4).

In ball games such as table tennis, in which a ball moving at high speed is hit, the visual system in the brain processes the motion information of the ball (5–7) to predict the arrival point and form a motor command for directing the racket there. Therefore, the quality of the visual information processing is an important factor in determining the quality of the physical movement (visuomotor response) that is subsequently performed. Visual sensitivity is known to vary daily, for example, contrast sensitivity has been reported to vary significantly in the middle range including optimal spatial frequency (8). Thus, day-to-day fluctuations in the visuomotor response may be ascribed to fluctuations in visual motion discriminability, but it is not clear whether or how they are related.

Brain information processing for seeing and hitting a moving ball is performed through the dorsal visual pathway in the visual system. The pathway includes the human motion-sensitive V5/MT+ complex (hMT+, the putative homolog of macaque MT) in the posterior bank of the superior temporal sulcus of the dorsal medial temporal cortex and the parietal cortex (9, 10). The hMT+ is a center for processing visual motion and generating visual motion sensation/perception (11). Interestingly, the superior visuomotor performance of athletes in response to visual motion is primarily related to visual perception (5).

Previous studies focusing on visual evoked potentials (VEPs) measured using electroencephalography (EEG) found that visual motion onset evokes a potential with negativity N2 around 150–200 ms above hMT+ and has been reported to reflect the activity relevant to motion perception and/or visual motion information processing (12–16). The faster visuomotor response by table tennis players and badminton players has been reported to correlate with the shorter latency of N2 (5, 6). In a series of studies on visual motion reaction performance, the latency to press a button in response to radial motion stimulus on a computer screen or a moving table tennis ball was evaluated as the visual motion reaction time (7). However, in a real ball game, not only quick reactions but also spatial accuracies of visual and motor information processing are required to hit the ball. Therefore, it is necessary to know how the spatiotemporal performance to reach the effector to a moving target is related to the visual motion discrimination performance. In addition, it is not clear whether the state of motion vision contributes to the performance in a continuous visuomotor response, such as in table tennis.

Visual motion direction discrimination (MDD) task using coherent motion by random dot kinematograms (RDK) as the visual stimulus has been performed as a functional measurement of hMT+ (17–20). The processing of motion changes throughout the visual hierarchy, from spatially restricted 'local motion' in the early visual cortex to more a complex large-field “global motion” in later stages. Since RDK forces the visual system to extract the global coherent direction of motion from local motion signals that have to be integrated over space and time before motion direction can be perceived (11, 21–23), it is suitable for investigating the function of hMT+. However, it is not clear how much the visual motion discrimination ability evaluated by RDK fluctuates daily or how much this ability contributes to the performance in the reach movement (intercept). Therefore, it remains unclear how daily fluctuations in visual motion discriminability affect continuous visuomotor performance. In particular, the effect of the fluctuations in motion vision on the continuous visuomotor performance such as the rally in table tennis is not known at all.

To investigate the performance in continuous visuomotor responses, a visuomotor task that continuously intercepts moving targets is required. However, when the racket in a sport like a table tennis is moved quickly and continuously, the influence of muscular factors, such as the legs and arms, becomes large, making it difficult to distinguish the factors of brain information processing.

Therefore, this study examined whether and how the day-to-day fluctuations in the continuous visuomotor response of table tennis athletes ascribe to the fluctuations in visual motion discriminability. To achieve this goal, we adopted a continuous visuomotor (CVM) task that can quantify the performance of continuously visual-based interceptive responses to fast-moving targets without effects from physical factors such as leg and arm strength (24). The visual motion discriminability was assessed in the MDD task, which required discrimination of the direction of a global coherent motion of target dots that move in the same direction on a PC monitor with a background of innumerable dots moving in random directions. The athletes performed the CVM task and the MDD task over 10 days, and then examined using correlation analysis whether and how the scores in the CVM and the MDD tasks covaried.

## Materials and methods

### Participants

Fourteen (mean ± SD: age = 20.4 ± 1.3 years, table tennis experience = 8.8 ± 2.4 years; 2 females; 1 left-handed) table tennis players took part in this study. Participant's competitive history and level in table tennis are shown in Table 1. All participants had normal or corrected-to-normal visual acuity. The study was approved by the ethics committee of the Graduate School of Medicine, Osaka University, and was conducted in accordance with the Declaration of Helsinki. Informed consent regarding the aim and experimental protocols of the present study was obtained in writing from all participants before participating in this study.

**Table 1:** Participant's competitive history and level in table tennis.

	**Years of experience**	**Competition levels**
sub1	8	/
sub2	9	Seven Universities Athletic Meet Single BEST8
sub3	10	Seven Universities Athletic Meet Doubles BEST32
sub4	10	/
sub5	13	Seven Universities Athletic Meet Doubles BEST16
sub6	6	Seven Universities Athletic Meet Doubles BEST32
sub7	9	Regional competition Single BEST 4
sub8	11	Seven Universities Athletic Meet Single BEST4
sub9	8	/
sub10	4	/
sub11	12	Seven Universities Athletic Meet Doubles BEST32
sub12	7	/
sub13	9	Seven Universities Athletic Meet Doubles BEST32
sub14	7	/

### Motion direction discrimination (MDD) task

**Figure 1:**
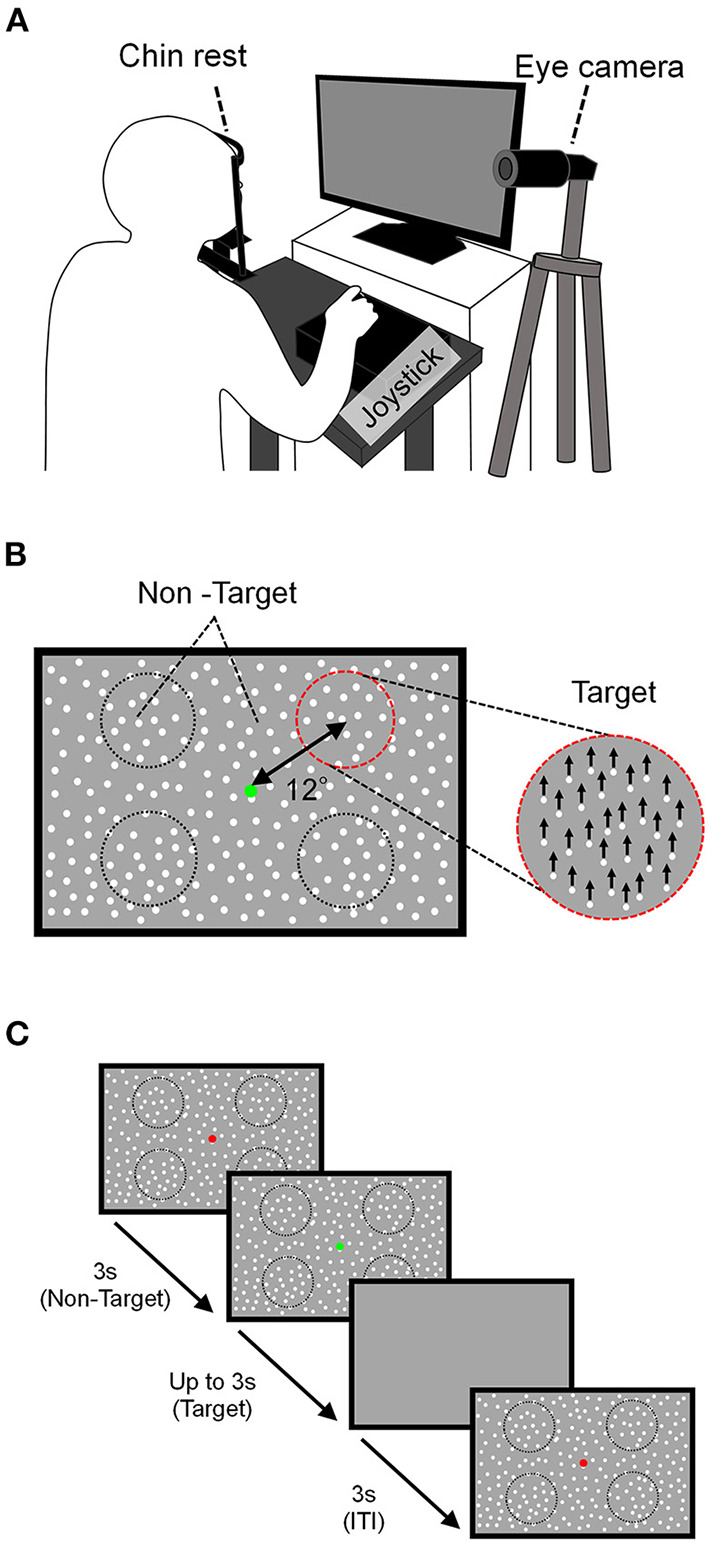
Schema of the MDD task. **(A)** The experimental setup. The participant placed his/her head on a chin rest 57 cm in front of the LC display and responded to the direction of movement of the target using a joystick. Right eye movements during the task were recorded using a USB camera. **(B)** Stimulation composition. The target area was a circle (dotted line) and was presented at four locations centered on a point 12° away from the fixation point. The motion direction of the target stimulus was either up, down, left, or right, which is upward in this example. **(C)** Task sequence. After non-targeted stimuli are presented with a red FP for 3 s, the color of the FP turned green, the target stimulus was presented for up to 3 s, and after 3 s of ITI, the next attempt began.

Visual motion discriminability was evaluated using the MDD task. Here, visual stimuli were generated using a custom-made program in Python and displayed on a liquid crystal (LC) display (Iiyama, Tokyo, Japan; resolution, 1920 × 1080 pixels; refresh rate, 100 Hz; mean background luminance, 30 cd/m^2^; screen size, 60 × 34° at a viewing distance of 57 cm). Participants sat about 57 cm in front of the LC display, and their heads were fixed on a chinrest (TKD-UK1, Namoto Trading Co., Ltd., Chiba, Japan) that positioned the center of the LC display to restrict their head movement. Participants responded to a visual stimulus using a joystick (JC-AS01BK, Elecom, Osaka, Japan). The participants' right eye movements during the task were recorded using a USB camera (Grasshopper3, Point Gray, Japan) and an eye-tracking system [iRecHS2, (25)] at 500 Hz (Figure 1A).

In the MDD task, the moving dots as the visual stimulus were presented on the display (Figure 1B). The dot size was 0.1° in diameter, the lifetime was 180 ms (18 frames), the density was 1.5 dots/deg and the speed was 15 deg/s. The visual stimulus was composed of target and non-target stimuli and a fixation point (FP) presented at the center of the display. The target stimulus contained dots moving in the same direction within a circular area (8° visual angle in diameter), and the ratio of dots moving in the same direction to all dots within the target stimulus was defined as the “motion coherence.” The target stimulus moved in one of four directions: upward, downward, rightward, and leftward. The target was presented in any one of the four circular areas that were set 12° away from the FP and located 45, 135, 225, and 315° visual angles counterclockwise from the upper right.

The target location was randomly changed to one of the four positions for each trial, and the entire display outside the target stimulus displayed dot stimuli (non-target stimulus) that moved in random directions with 0% motion coherence. The MDD task began by displaying non-targeted stimuli across the display concurrently with a red FP (Figure 1C). After 3 s, the color of the FP turned green, the target stimulus was presented for up to 3 s, and then all stimuli on the display disappeared for 3 s (ITI; intertrial interval). Participants were instructed to keep gazing at the FP during the task and to answer the motion direction of the dots in the target by tilting the joystick toward the discriminated direction as quickly as possible with the dominant hand after the FP turned green. Trials in which participants tilted the joystick toward the correct direction were defined as a “correct trial,” and tilting the joystick toward the incorrect direction or no response in 3 s was defined as an “incorrect trial.” When participants responded, a feedback-sound was given.

### Continuous visuomotor (CVM) task

**Figure 2:**
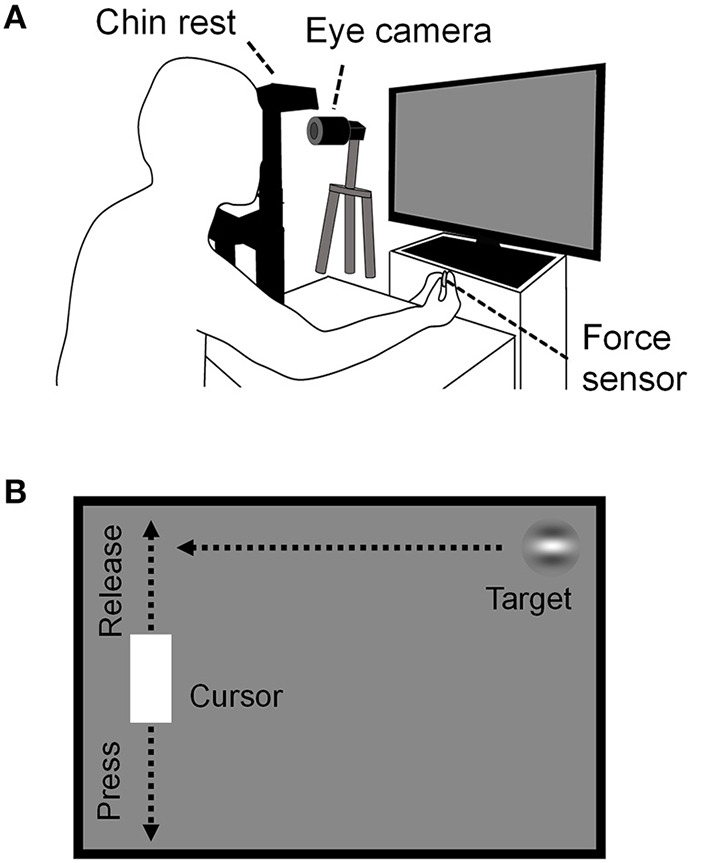
Schema of the CVM task. **(A)** The experimental configuration is the same as in Figure 1, except that the participants prehend the force sensor with the thumb and index finger of the dominant hand. Left eye movements during the task were recorded using a USB camera. **(B)** Outline of the CVM Task. The target moved in a constant speed horizontally. The cursor moved in the Y-axis direction only, and its position corresponded to the magnitude of the prehensile force given to the force sensor.

Continuous visuomotor performance was evaluated using the CVM task (24). Here, visual stimuli were generated using a custom-made program in Python and displayed on the same LC display used for the MDD task. Participants sat about 57 cm in front of the LC display and their heads were fixed on a chinrest that was positioned at the center of the Y-axis and one-thirds of the X-axis from the left side of the LC display. The participants grasped the force sensor (USL06-H5-50N, Tec Gihan Co., Ltd., Japan; sampling rate, 1 KHz;) with the thumb and index finger of their dominant hand. Their left eye movements during the task were recorded at 500 Hz using the same USB camera and eye-tracking system for the MDD task (Figure 2A).

A Gabor patch with horizontal grating (target, diameter, 90 pixels; spatial frequency, 1.5 cycles/deg; contrast, 50%) and a white rectangle [cursor, height, 180 pixels; width, 96 pixels; RGB (1,1,1)] were presented on the LC display (Figure 2B). The target appeared from the right edge of the LC display and moved at a constant speed horizontally. The target speeds were 1000, 2000, 3000, 4000, 4500, 5000, 6000, and 7000 pixels/s, which corresponded to 29, 57, 86, 114, 127, 144, 172, and 203 deg/s in visual angular speed. The angular speed was calculated by dividing the angle formed by the two vectors from the midpoint of both eyes to the right end of the LC display and to the left end of the cursor by the target arrival time (TAT). TATs were defined as the times from the moment of the target appearance to the arrival at the horizontal position of the cursor, and they for each target speed were 1563, 782, 521, 391, 347, 313, 261, and 223 ms, respectively. The task duration was set to 30 s for all target speed conditions in order to eliminate effects such as fatigue on task performance as much as possible because the task requires high concentration to perform. The number of trials for each target speed was 18, 37, 54, 70, 80, 86, 103, and 119, respectively. As soon as the target hit the cursor or reached the left edge of the display, the next target appeared at the random height on the right edge of the display. The cursor was presented at 240 pixels from the left edge of the LC display and could only move vertically (i.e., along the Y-axis). The position of the cursor on the Y-axis corresponded to the magnitude of the prehensile force given to the force sensor: top of the display at minimal prehensile force and bottom of the display at 30% maximum prehensile force. Therefore, weakening (releasing) the prehensile force raises the cursor, and strengthening (pressing) lowers the cursor. The participants were instructed to hit the target with the cursor without moving their head and hand positions, but no instruction was given for their eye movement. They were able to freely move their eyes anywhere within the LC display during the task. Trials in which participants hit the target were defined as “hit trials” and those in which they failed to hit the target were defined as “miss trials.”

### Experimental protocol

**Figure 3:**
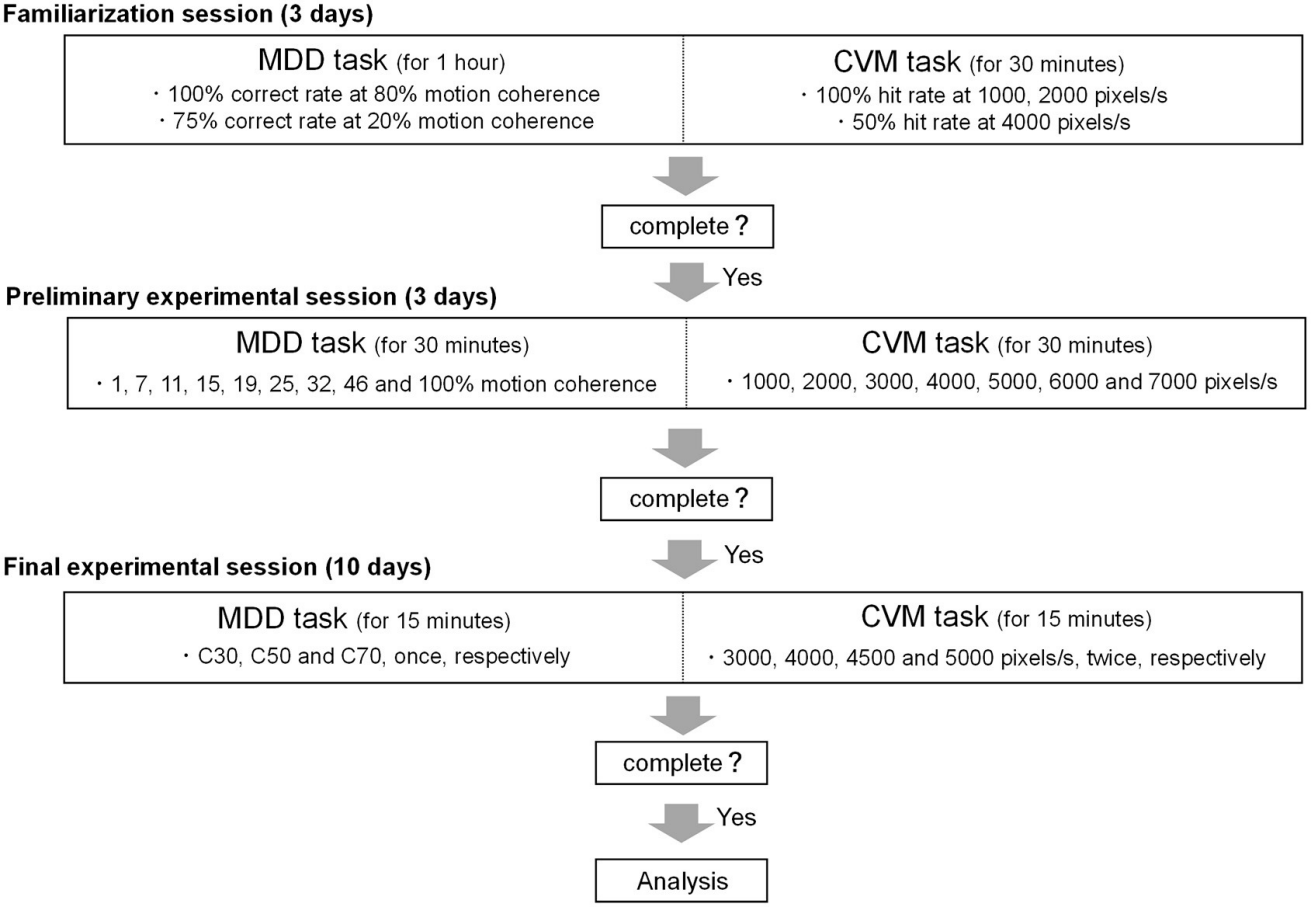
Schematic overview of the experimental protocol. The study was composed of a familiarization session (3 days), a preliminary experimental session (3 days) and a final experimental session (10 days).

In this study, participants conducted the MDD and CVM tasks multiple times, so it was necessary to minimize the effect of adaptive learning caused by task repetition on task performance. In addition, all MDD tasks should be performed at the same level of difficulty across individuals, but visual motion discriminability varies between individuals. Therefore, it was also necessary to set a task difficulty level for each participant. For the above reasons, this study was composed of a familiarization session (for 3 days), a preliminary experimental session (for 3 days), and a final experimental session (for 10 days) (Figure 3).

In the familiarization session, participants conducted the MDD task until the ratio of correct trials to a total number of trials (correct rate) reached 100% and 75% for a target motion coherence of 80% and 20%, respectively. Participants conducted the CVM task at 7 target speeds (1000, 2000, 3000, 4000, 5000, 6000, and 7000 pixels/s) until the ratio of hit trials to the total number of trials (Hit rate) reached 100% at 1000 and 2000 pixels/s and 50% at 4000 pixels/s. The times of the familiarization session of the MDD and CVM tasks were 1 h and 30 min per day, respectively.

**Figure 4:**
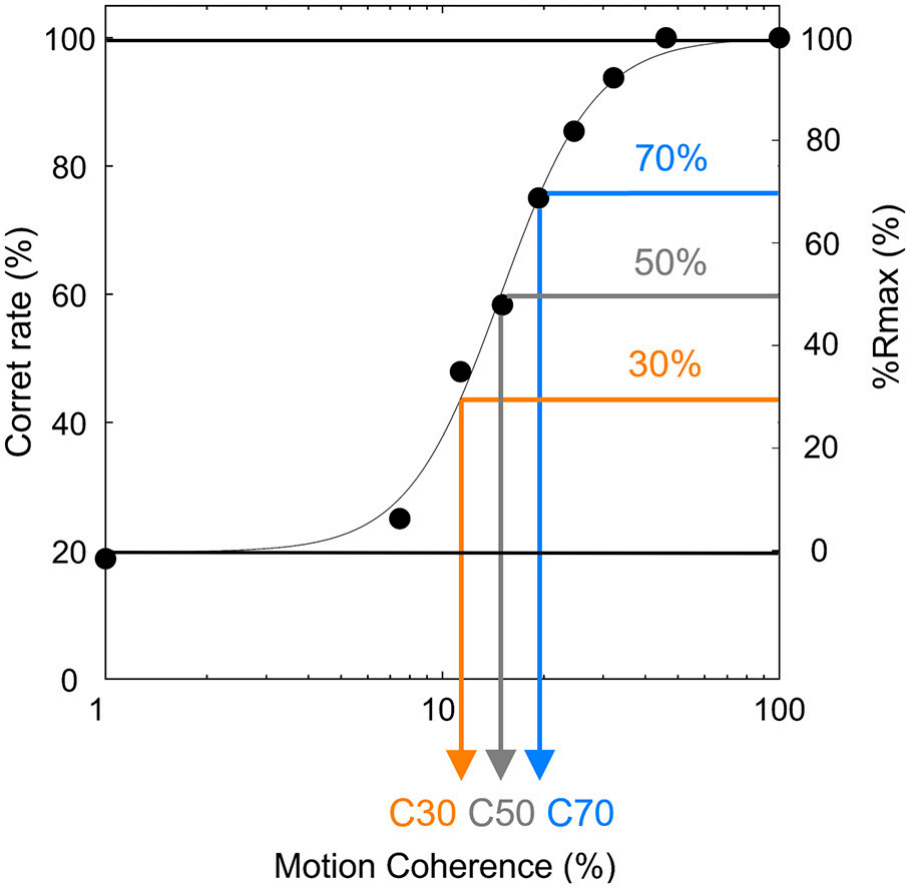
Typical result of a preliminary experiment session of the MDD task. The difference between the maximum correct rate and minimum correct rate was set to 100% (Rmax), and motion coherence values with 30, 50, and 70% Rmax (defined as C30 (orange), C50 (gray), and C70 (blue), respectively) were calculated.

In the preliminary experimental session, participants conducted the MDD task at 9 motion coherence conditions (1, 7, 11, 15, 19, 25, 32, 46, and 100%). The number of trials for each condition was 16 trials, and participants conducted all conditions per day. The average value of the correct rate for 3 days was fitted to a sigmoid curve using the Naka-Rushton function (26), and the difference between the maximum correct rate and the minimum correct rate was set to 100% (Rmax). Since the difficulty of motion direction discrimination may affect the magnitude of day-to-day fluctuations, we tested 30, 50, and 70% Rmax conditions where ceiling/floor effects caused by too-easy/too-difficult conditions could be avoided. These Rmaxs were calculated as the motion coherence values being used in the final experimental session and defined as C30, C50, and C70, respectively (Figure 4). The time of the preliminary experimental session was 30 min per day.

In the final experimental session, participants conducted the MDD task at the 3 target coherence conditions (C30, C50, and C70) once daily and CVM task at 4 target speed conditions (3000, 4000, 4500, and 5000 pixels/s) twice daily. A recent study using the CVM task (24) found that RCA is controlled in a feedforward manner based on the initial visual information obtained, but is also corrected in a feedback manner with the new visual information obtained after ending saccade at the target. However, visual feedback control was limited to a target speed of 3000 pixels/s, and RCA was mainly generated by feedforward control above 5000 pixels/s. It suggests that the target speed affects the motor control for the cursor movement. Therefore, a range of speeds encompassing them (3000 - 5000 pixels/s) was set in this study. The time of the final experimental session of the MDD and CVM tasks was 15 min each per day. All participants took part in all sessions and conducted the CVM task after the MDD task. The final experimental session was scheduled to be the same time (±28.9 min) for a participant. Participants were instructed to abstain from alcohol and caffeine for 24 h before each experimental day.

### Data analysis

Data for the MDD and CVM tasks in each participant were normalized as z-scores for each experimental day. The z-score of the CVM task was called “the CVM task score” and was used as an index of CVM task performance. In the MDD task, the correct answer rate (accuracy) and the mean reaction time of correct trials (speed) were calculated for each measurement day as the visual motion discriminability. The correct rates and mean reaction times were z-scored, respectively, and then, the mean values of both parameters were calculated for each measurement day and defined as the total visual motion discriminability of each day, and referred to as “the MDD task score.”

**Figure 5:**
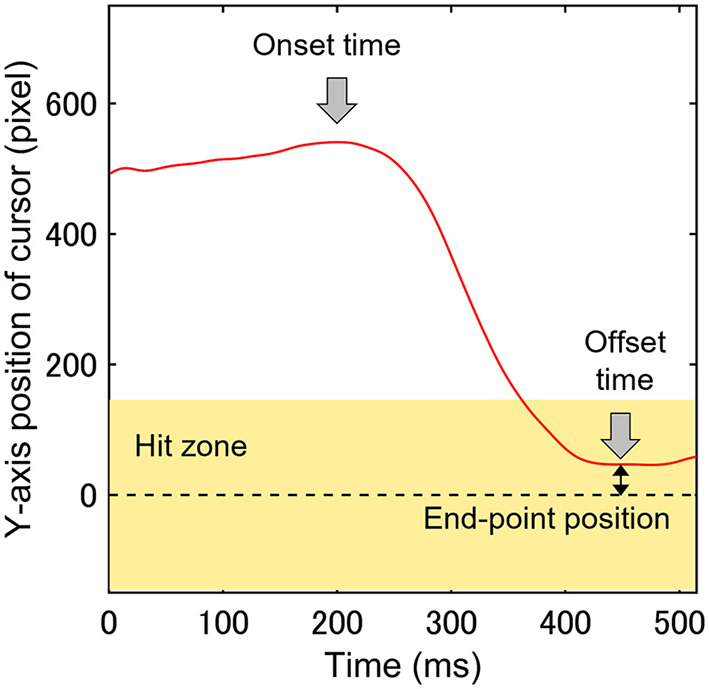
A single trajectory of cursor movement along the Y-axis was used to analyze three parameters (RCA onset time, offset time, and end-point position). The light yellow band indicates the Hit zone, and the zero value of the Y-axis is the center of the target.

In the CVM task, the raw data for the cursor and eye movements were filtered (fourth-order Butterworth low-pass filter with a 30-Hz cut-off and 0-time shift using MATLAB). The Y-axis range in which the distance between the target and cursor centers was less than the sum of the target radius and half the vertical length of the cursor was called the “Hit zone” where the target and the cursor collide. Mathematically, the Hit zone length, defined as the distance between the center of the target and the center of the cursor, is 135 pixels or less. However, filtering to remove high-frequency noise in the electrical signal from the force sensor that determines cursor position can introduce an error of less than about 10 pixels, so 145 pixels were chosen to account for that. Figure 5 shows the trajectory of the cursor along the Y-axis of the display in a single trial. Participants typically moved the cursor quickly to the Hit zone (light yellow band in Figure 5) after the target appearance to hit the target. We referred to this movement as the rapid cursor approach (RCA). The method of calculating the parameters related to the RCA followed the method of Aoyama et al. (24). The RCA occurrence rate was calculated as the incidence percentage of RCA relative to the total number of trials. To evaluate the temporal characteristics of the RCA, the start and end times of the RCA were defined as the RCA onset time and the offset time, respectively (Figure 5). At the time of the RCA offset, the Y-axis absolute distance between the centers of the Hit one and cursor was defined as the RCA end-point position and evaluated as the spatial accuracy of the RCA (Figure 5). The following trials were excluded from the analysis based on the criterion of a previous study using the same CVM task (24): (1) trials with no requirement for the RCA; that is, the cursor was in the Hit zone at the beginning of each trial; (2) trials in which the RCA onset time was < 80 ms, because at least an 80 ms onset latency is needed for a visually-triggered body movement (27); and (3) trials in which the cursor moved in the direction opposite of the target. In the CVM task, the ratio of hit trials to the total number of trials (Hit rate), the RCA occurrence rate, the mean of RCA onset time of total trials (RCA onset time), and the mean of the RCA end-point position of total trials (RCA end-point position) on each day were calculated as the visuomotor performance. The eye movements in the CVM task were analyzed with a focus on movement in the Y-axis direction in this study.

### Statistical analysis

For each task, to examine the difference in mean scores between target conditions, we first used the Shapiro-Wilk test to check whether the raw data had a normal distribution. ANOVA was used for data with normal distribution and Friedman's test was used when a non-normal distribution was found. On the other hand, the difference between experimental conditions in variance (SDs) of task scores over 10 days for each task was tested by Bartlett's test. In comparison with the usage rate for capturing the target between the right-side visual field and the left-side visual field from the emersion of the target to the onset of the RCA in the CVM task, the Wilcoxon signed-rank test was used, since the data had non-normal distribution. The correlation coefficient between the MDD task scores and the CVM task-related scores (the Hit rate, RCA occurrence rate, RCA onset time, and RCA end-point position) obtained over 10 days from the final experimental session was calculated. It was calculated using Pearson's correlation for normally distributed data and Spearman's correlation for non-normally distributed data. The significance level was set at 5% for all statistical tests. We performed G-power analysis to examine the confidence of correlation analysis, that is, whether the correlation coefficient is statistically meaningful, and confirmed the confidence for all the correlation analyses.

## Results

### Overall features of performances in the MDD and CVM tasks

We first performed a statistical test on the difference in the mean value of 10 days between experimental conditions in both MDD and CVM task scores. In the MDD task, there was a significant difference among coherence conditions (*p* < 0.001). In the CVM task, a statistical difference in the Hit rate was observed among target speed conditions (*p* < 0.001). Next, we examined differences in the variance (SDs) of 10 days between experimental conditions in both MDD and CVM task scores. In the MDD task, there was no difference in the variance of correct rate among C30, C50, and C70 conditions, but reaction time showed a significant difference among the conditions (*p* < 0.001). In the CVM task, the variance of the Hit rate was statistically significant among target speed conditions (*p* < 0.001).

### Daily fluctuations in visual motion discriminability and continuous visuomotor performance

**Figure 6:**
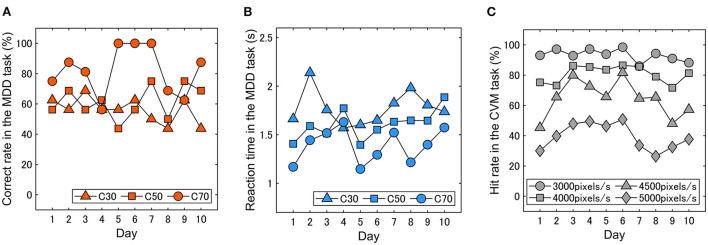
Typical results in the day-to-day fluctuations in **(A)** correct rate and **(B)** reaction time in the MDD task and **(C)** hit rate in the CVM task. The visual motion discriminability and continuous visuomotor performance fluctuated daily.

We examined whether and how much the performances in visual motion discriminability and continuous visuomotor fluctuate day to day. Figures 6A–C show typical results from a participant, showing that the performance in the two tasks changes daily and that the magnitude of the fluctuations differs depending on the stimulus conditions. The magnitude of day-to-day fluctuation was evaluated as the standard deviation (SD) of the data. In the MDD task, the SDs of the correct rate for each condition were 8.3% (C30), 10.5% (C50) and 16% (C70), and those of the reaction time were 0.18 s (C30), 0.15 s (C50) and 0.18 s (C70). In the CVM task, the SDs for each condition were 4.0% (3000 pixels/s), 5.7% (4000 pixels/s), 20.0% (4500 pixels/s) and 8.8% (5000 pixels/s).

### Gross association in performance fluctuations between the MDD and CVM tasks

Next, we investigated whether there is a relationship between the daily fluctuations in the MDD and CVM task scores. The significance levels were corrected by the Bonferroni method and set to 5%. Table 2A shows a matrix of correlation coefficients that were calculated from the data of all participants for the combination of three motion coherent conditions (C30, C50, C70) in the MDD task and four target speed conditions (3000, 4000, 4500, 5000 pixels/s) in the CVM task. A statistically significant and moderate correlation (*r* = 0.53, *p* < 0.001) was observed for C70 and 5000 pixels/s, and a significant but weak correlation was found for C30 and 4000 pixels/s (*r* = 0.27, *p* < 0.05) and for C50 and 4500 pixels/s (*r* = 0.27, *p* < 0.05).

**Table 2:** Correlation coefficients for the scores in the (A) MDD task and CVM task, (B) MDD task for a target that appeared in the right-side visual field and CVM task, and (C) MDD task for a target that appeared in the left-side visual field and CVM task.

**A**					
		**CVM task (pixels/s)**			
		**3000**	**4000**	**4500**	**5000**
	C70	r = 0.14	r = 0.23	r = 0.19	r = 0.53^**^
MDD task	C50	r = 0.12	r = 0.2	r = 0.27^*^	r = 0.21
	C30	r = 0.01	r = 0.27^*^	r = 0.09	r = 0.09
**B**					
		**CVM task (pixels/s)**			
		**3000**	**4000**	**4500**	**5000**
	C70	r = 0.19	r = 0.25^*^	r = 0.15	r = 0.58^**^
MDD task	C50	r = 0.14	r = 0.16	r = 0.19	r = 0.12
	C30	r = 0.06	r = 0.22	r = 0.1	r = 0.06
**C**					
		**CVM task (pixels/s)**			
		**3000**	**4000**	**4500**	**5000**
	C70	r = 0.02	r = 0.09	r = 0.15	r = 0.21
MDD task	C50	r = 0.01	r = 0.15	r = 0.2	r = 0.2
	C30	r = −0.07	r = 0.22	r = 0.04	r = 0.11

* *p* < 0.05,

** *p* < 0.001.

### Relationship between visuomotor control and gaze behavior in the CVM task

**Figure 7:**
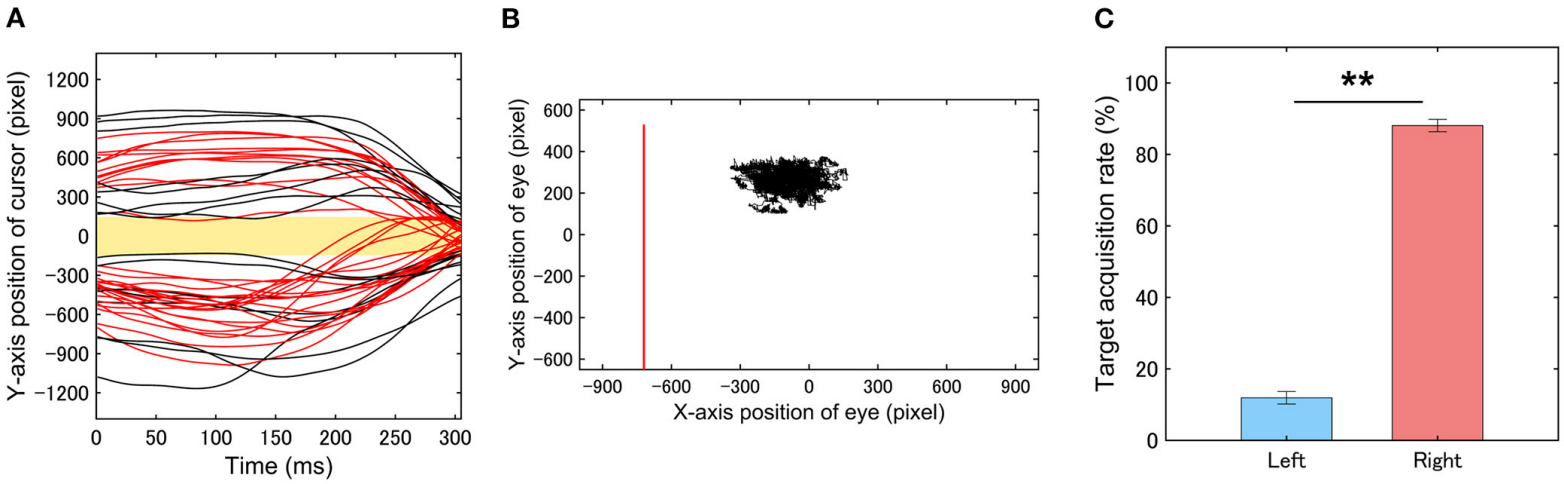
**(A)** Typical examples of trial trajectories of the cursor movement along the Y-axis. Red and black lines indicate Hit and Miss trials, respectively. Light yellow rectangle indicates ‘'Hit zone”. **(B)** Typical examples of eye movements while performing the CVM task. Red lines show the cursor movement. **(C)** Target acquisition rate of the left- and right-side visual fields from the appearance of the target to the onset of the RCA. Participants acquired the target information in their right-side visual field predominantly to start the RCA. Error bars are SEM. **p < 0.01.

Figure 7A shows typical examples of temporal change in the cursor position on the Y-axis of the LC display under a target speed condition of 5000 pixels/s in relation to the Hit zone. RCA was observed almost at all the trials with a latency longer than about 150 ms after the target appearance in the CVM task. The participants placed their gazes around the center of the width of the LC display throughout the CVM task, suggesting that there is a bias in the visual field to acquire the target information (Figure 7B). To confirm this point, we examined how long the target was captured in the left- or right-side visual field by analyzing the horizontal position of the gaze relative to the target from the appearance of the target to the onset of the RCA (Figure 7C). The target was mostly on the right side of the participant's gaze (left, 11.9 ± 1.7%, right, 88.1 ± 1.7%), and the difference between the left and right sides was statistically significant (Wilcoxon signed rank test, *p* < 0.001). This finding indicates that the target information essential for the success of the CVM task was obtained from the right-side of the visual field.

### Relationship between visual motion discriminability in the TAV hemifield field and continuous visuomotor performance

**Figure 8:**
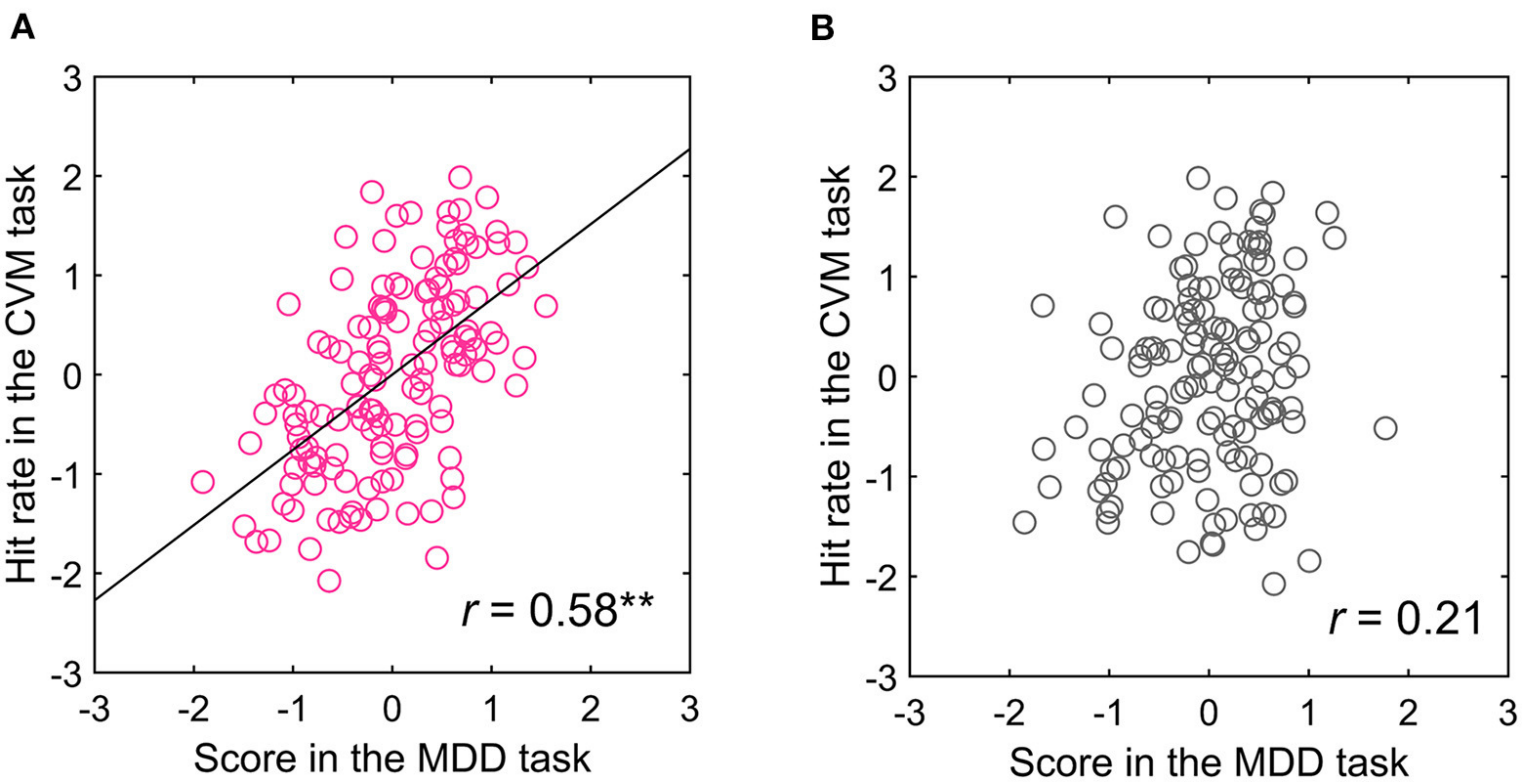
Relationship between the **(A)** TAV and **(B)** non-TAV hemifield scores in the MDD task at C70 and CVM task at 5000 pixels/s. **p < 0.001.

Because the acquisition of target information in the CVM task was extremely biased toward the right-side visual field and MT neurons have receptive fields in the contralateral visual field (28), hMT+ in the left hemisphere is mainly involved in the CVM task. If the left hMT+ contributed to the CVM task score, then the score in the right-side visual field in the MDD task will more strongly correlate with the CVM task score. Therefore, we separated the trials for targets presented in the right- and left-side visual fields in the MDD task and recalculated the correlation coefficients between the scores in the MDD and the CVM task (Tables 2B,C). Notably, one participant acquired the target information from the left field of view in the CVM task. Therefore, the visual hemifield acquiring the target information during CVM was defined as target-acquired visual (TAV) hemifield, and the TAV hemifield and the opposite (non-TAV) hemifield were analyzed separately. The one participant with the left TAV hemifield was analyzed for the relationship of the MDD task score in the left visual hemifield with the CVM task score. There was a significant and moderate correlation between C70 and 5000 pixels/s (*r* = 0.58, *p* < 0.001) in the TAV hemifield stimulated condition in the MDD task (Table 2B), but none (*r* = 0.21, *n.s*.) in the non-TAV hemifield stimulated condition (Table 2C). Figures 8A,B show scatter plots for the Hit rates in the CVM task at 5000 pixels/s against the MDD task score at C70.

The visual hemifield-dependent relationship between the MDD and CVM task scores suggests that visual motion discriminability evaluated using RDK is one of the determinants of the performance in the visuomotor response to a moving target. However, hemispheric asymmetries need to be considered before making this conclusion. For example, directing attention toward a visual stimulus has been reported to result in more bilateral representations (less contralateral bias) in the right than the left hemisphere for hMT+ (29). The attention-based bi-lateralization of the receptive field attenuates the relationship between the contralateral (left) visual field and the right hMT+, which may be responsible for the visual hemifield-dependent correlation in the MDD and CVM task scores. Also, although sleep observed in the unilateral hemisphere is not known to occur in humans, if there are factors that affect the unihemispheric (30), the primary motor cortex and visual cortex localized in the same hemisphere may exhibit spurious correlations in exerted performance levels without a direct functional connection.

**Figure 9:**
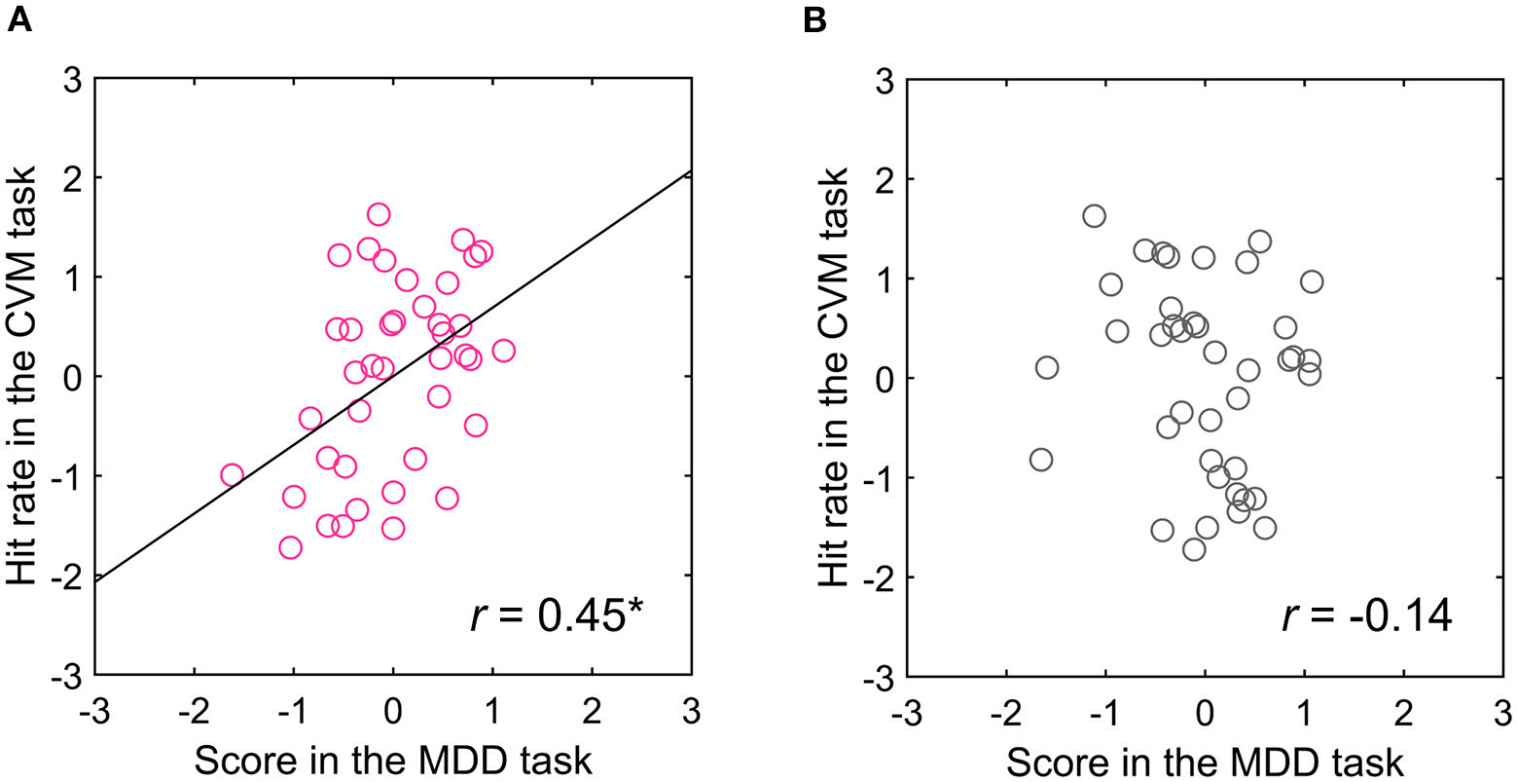
Relationship between the **(A)** TAV and **(B)** non-TAV hemifield scores in the MDD task at C70 and hit rate in the CVM task at 5000 pixels/s for the target moving from left to right on the LC display. *p < 0.05.

To examine these possibilities, we conducted an opposite motion direction version of the CVM task, where the target moves from left to right on the LC display. Even in this condition, the visual hemifield-dependent correlation between the MDD and CVM task scores was observed (*N* = 4, *r* = 0.45, *p* < 0.05) (Figure 9). The target was mostly on the left-side of the participant's gaze (left, 96.9 ± 0.9%, right, 3.1 ± 0.9%), and the difference between the left and right sides was statistically significant (Wilcoxon signed rank test, *p* < 0.001). So, the left-side visual field was the TAV hemifield for all participants.

In the normal target motion direction version, there was one participant who used the left-side visual field to get the target information in the CVM task, which showed a stronger correlation of the CVM task score with the MDD task score for the left-side visual field stimulation. We also compared the difference between the right- and left-side visual hemifields in the MDD task score and day-to-day fluctuation (SD), but found none in MDD task accuracy (right, 14.6%; left, 13.1%; both 11.0%) or reaction time (right, 0.2s; left, 0.2s; both, 0.2s).

### Participant's table tennis competition history and visual hemifield-dependent correlation with MDD and CVM task scores

**Figure 10:**
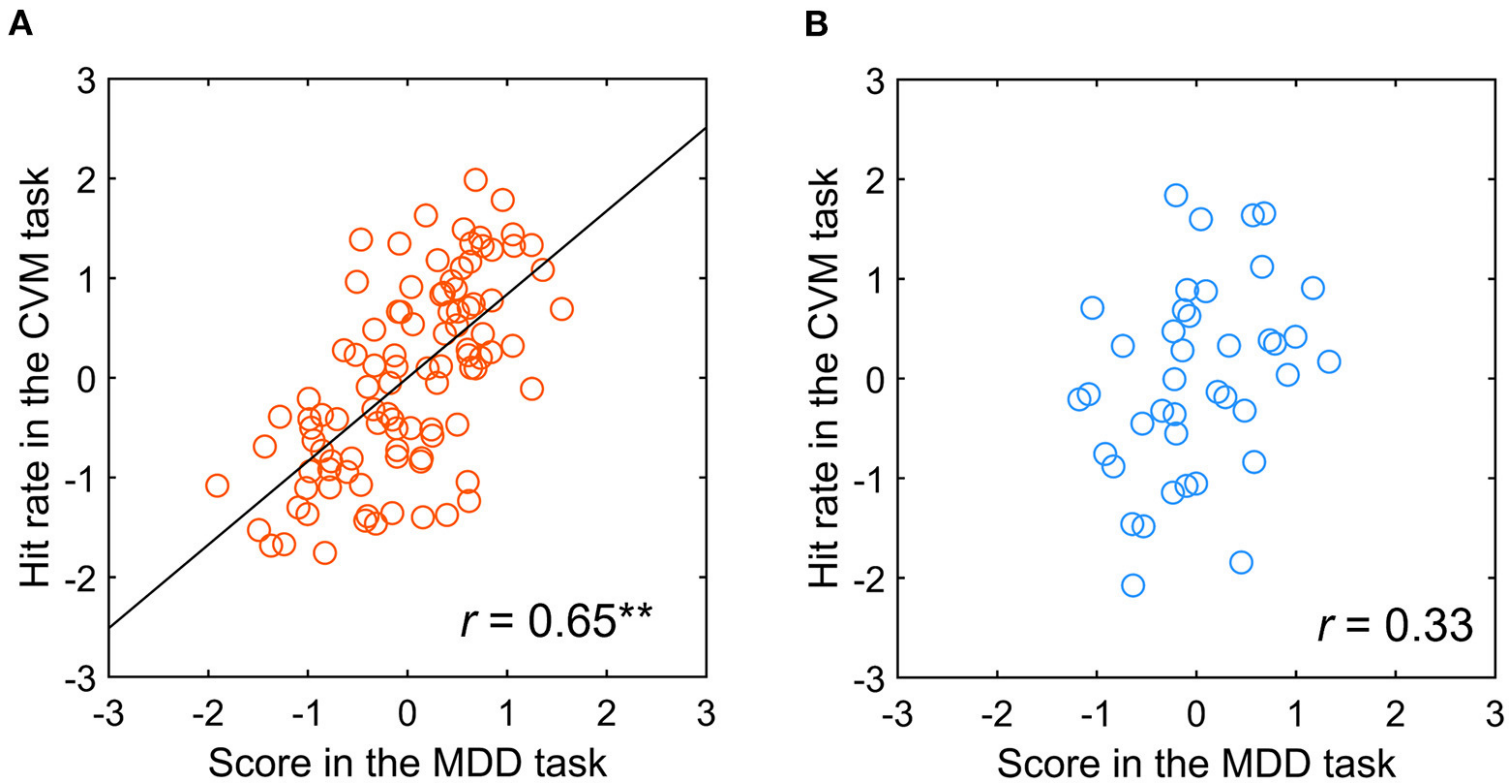
Relationship between the scores in the MDD task at C70 for the TAV hemifield and the CVM task at 5000 pixels/s by participants with **(A)** more than 8 years of competitive experience and **(B)** < 8 years. **p < 0.001.

A table tennis player who has more experience in converting the visual information of motion into physical body movement may have a stronger functional connection between the two information processing systems. Therefore, we examined the percentage of participants with a significant correlation between the MDD and CVM task scores, finding that a high percentage was observed in the participants having more than 8 years of competition history in table tennis. So, correlation analysis in a population level was performed for two participant groups divided by the 8 years history. In the participants having more than eight (*N* = 10), the correlation was significant between both tasks (*r* = 0.65, *p* < 0.001). On the other hand, for participants with < 8 years of experience (*N* = 4), none showed a significant correlation between the two tasks (*r* = 0.33, *p* = 0.45). Figures 10A,B shows scatter plots of the Hit rate in the CVM task at 5000 pixels/s and the MDD task score for the two groups. The difference between the two groups may be due to differences in the Hit rate of the CVM task, and the correct answer rate and reaction time of the MDD task. Therefore, when we performed a significant difference test on the mean value and variance of those raw data, no significant difference was observed in any of them.

### Relationship between visual motion discriminability in the TAV hemifield in the CVM task and spatiotemporal characteristics of the RCA

**Figure 11:**
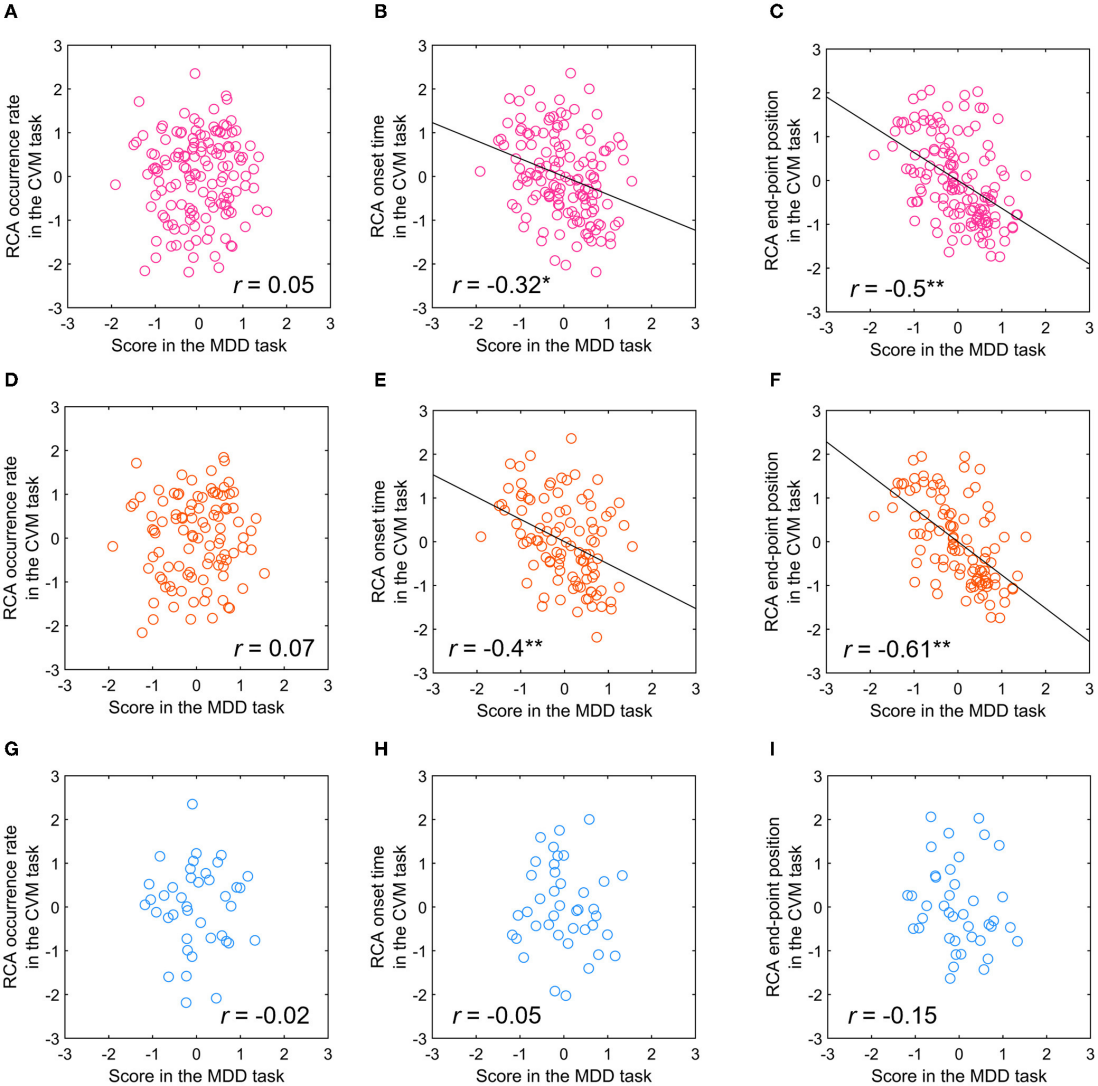
Relationship between the scores in the MDD task at C70 for the TAV hemifield and RCA occurrence rate, RCA onset time, and RCA end-point position of the CVM task at 5000 pixels/s. **(A–C)** All participants. **(D–F)** Participants with more than 8 years of competitive experience. **(G–I)** Participants with < 8 years of competitive experience. *p < 0.01, **p < 0.001.

From the viewpoint of the day-to-day co-fluctuations, we examined the relationship between the MDD task score and RCA-related parameters (the occurrence rate, the onset time, and the endpoint position) in the CVM task. For all participants, the MDD task score showed a significant but weak correlation with the RCA onset time (*r* = −0.32, *p* < 0.01) and significant and moderate correlation with the RCA end-point position (*r* = −0.5, *p* < 0.001), but no correlation with the RCA occurrence rate (*r* = 0.05, *n.s*.) (Figures 11A–C). For the participants with at least 8 years of experience, the MDD task score again showed a significant but weak correlation with the RCA onset time (*r* = −0.4, *p* < 0.001) and a significant and moderate correlation with the RCA end-point position (*r* = −0.61, *p* < 0.001), but no correlation with the RCA occurrence rate (*r* = 0.07, *n.s*.) (Figures 11D–F). On the other hand, for the participants with < 8 years of experience, no significant correlation with the RCA onset time (*r* = −0.05, *n.s*.), the RCA end-point position (*r* = −0.15, *n.s*.) or the RCA occurrence rate (*r* = −0.02, *n.s*.) was observed (Figures 11G–I).

## Discussion

In the present study, we investigated whether and how day-to-day fluctuations in the continuous visuomotor score (the Hit rate of the CVM task) are due to day-to-day fluctuations in visual motion discriminability (the MDD task score). The main results of this study were: (1) visual motion discriminability and continuous visuomotor scores fluctuate daily, (2) there exists a visual hemifield-dependent correlation between the daily fluctuations in the two task scores, (3) this correlation was observed only in participants who had extensive experience at table tennis, and (4) experienced participants also showed correlations between visual motion discriminability and the RCA onset time and the end-point position but not with the RCA occurrence rate.

### Day-to-day fluctuations in the MDD and CVM task scores

Both scores of the MDD and CVM tasks varied day-to-day and covaried over 10 days for specific task conditions. There are some reasons for the covariation of the scores, such as arousal level. For example, high arousal state leads to not only high perceptual detection/discrimination ability of visual stimulus but also high motor output, which makes a pseudo-correlation between the two tasks. However, it seems unlikely because the relationship between the MDD and the CVM task scores was dependent on the visual hemifield, and a significant correlation was found in the experimental conditions in which target visual information was processed in the same cerebral hemisphere in the two tasks. It cannot be explained by the pseudo-correlation between independent functions of the brain due to the fluctuation of the global brain state. Therefore, we believe that the day-to-day fluctuation of the score of the continuous visuomotor score reflects that of visual motion discrimination ability. We speculate that visual regions in the brain with visual field maps and neurons that are selective in the motion direction of stimulus may have contributed to both task scores, resulting in covariation between task scores.

The reason why day-to-day covariation between both task scores was only observed between C70 for the MDD task and a target speed of 5000 pixels/s for the CVM task should be considered from two perspectives. First, the motion coherence in the MDD task corresponds to the signal intensity to the visual motion processing system, and low coherence makes the motion itself difficult to detect. However, in the CVM task, the direction of motion can be clearly perceived at any target speed, which is close to the high coherence condition in the MDD task, which may explain why the significant correlation was observed only in the C70 condition. Second, in terms of target speed in CVM, a target speed of 5000 pixels/s forces participants to process visual information processing in a very short time, perhaps making this process a bottleneck for visuomotor responses. Therefore, the state of the information processing process of moving targets on the task day is easily reflected in the visuomotor score, which may have resulted in a correlation with the MDD task score requiring motion processing. However, further study is needed to clarify this point.

### Day-to-day fluctuations in the MDD task score

This study demonstrated that the visual motion discriminability evaluated by RDK contributes to continuous visuomotor performance from the viewpoint of day-to-day fluctuations in the visual and visuomotor functions. The central region of motion perception in humans is in the posterior bank of the superior temporal sulcus of the dorsal medial temporal cortex (hMT+) (31–33). In human functional magnetic resonance imaging (fMRI) studies, it has been consistently reported that larger blood oxygen level-dependent (BOLD) responses occur in the parietal cortex, especially in the human motion-sensitive V5/MT+ complex (putative homolog of macaque MT) as well as in the parietal cortex (10), when targets are detected more than when they are missed (34–36). A monkey study reported that, under the motion coherence condition where the correct answer rate for the motion direction discrimination task was about 50:50, the visual response magnitude of a single MT neuron was able to predict the success or failure of the task (37). It strongly suggests the neuronal response in area MT contributes to perceptual decision-making on motion direction discrimination. Therefore, the day-to-day fluctuations in visual motion discriminability in this study may reflect the day-to-day fluctuations of reactivity in area hMT+. Here, a question arises as to why the visual motion detection/discrimination ability fluctuates from day to day but it remains unknown. Although various factors, such as arousal level, attentional function, physical and psychosocial stress, and emotional factors, need to be considered to affect visuomotor function (38, 39), there are reports indicating the involvement of material factors. Daily fluctuation in perceptual contrast sensitivity has been reported to show a dependency for spatial frequency, in which the most sensitive spatial frequency was varied (8). Interestingly, our recent animal studies found that neuromodulators such as acetylcholine, noradrenaline, and serotonin in the rat brain modulate contrast sensitivity with the same spatial frequency dependency reported by Andrade et al. (40–42). Therefore, day-to-day fluctuation in the level of neuromodulators in the brain is considered one of the candidate factors causing day-to-day fluctuation in perceptual visual motion detection ability.

### Day-to-day fluctuations in the CVM task score

To clarify the reason for day-to-day fluctuation in the CVM task scores, it is necessary to understand how cursor movement is controlled during the CVM task. Our recent study (24) using the CVM task revealed that the RCA is controlled by visual feedback information acquired after a predictive saccade to the target prior to the ending of the RCA. This suggests that functional fluctuations in the eye movement system can contribute to fluctuations in the CVM task score. However, the visual feedback control in Aoyama's study was observed only at a middle target speed of 3000 pixels/s, and a feedforward motor control based on visual information obtained initially was reported to make the RCA at a high-speed range of 5000 pixels/s or more. In this study, as shown in Figure 7B, the participants rarely performed saccade movements to direct their gaze to the target, suggesting that the eye movement itself hardly contributed to the task score *per se* and the day-to-day fluctuation. This means that the visual information acquired at the time of target appearance contributes greatly to the CVM task score and that the early visual system which contributes to motion information processing in the visual hemifield that acquires that information plays an important role. Therefore, the contribution of this early visual system function to the scores of the MDD task and the CVM task is considered to be a major reason for a co-fluctuation between both task scores.

However, the statistical results on the variance of the CVM task scores indicated that there is a significant difference between the target speed conditions and that the variance at 5000 pixels/s was the largest among the conditions. Therefore, it may be possible that such a large variance is one of the reasons why a significant correlation between two task scores was observed only at 5000 pixels/s. Further study is needed to clarify this point.

### Relationship between visual motion discriminability and continuous visuomotor response

This study revealed that visual motion discriminability (the MDD task score) and continuous visuomotor response to a moving target (the CVM task score) were co-fluctuated over 10 days. Moreover, the relationship showed a visual hemifield dependency, with a significant correlation found only between the fluctuations in the MDD task score in the hemifield where the target information was obtained in the CVM task. This finding suggests that early visual areas with receptive fields in the contralateral visual hemifield and with sensitivity to visual motion contributed to both tasks and are involved in the fluctuations. Of the two major visual streams in the brain, the dorsal visual pathway provides visual information for behavioral guidance (43), and the hMT+ is a promising candidate. Repetitive transcranial magnetic stimulation (rTMS) to the hMT+ while reaching and intercepting a downwardly moving visual target that follows an unpredictable curved trajectory displayed on a vertical screen affected both the interception timing and spatial position (44). Therefore, hMT+ may be involved in both the temporal control (45–47) and spatial control (44) of visually guided arm movements. Consistent with this hypothesis, the MDD task scores in the present study showed a significantly negative correlation with the RCA onset time and endpoint position in the CVM task. The RCA onset time and endpoint position reflect the speed and spatial accuracy of visual information processing, respectively, and small values of both parameters indicate fast processing speed and high spatial accuracy. Therefore, our findings provide indirect evidence that motion discriminability related to the hMT+ function contributes to spatiotemporal control of the visuomotor reaction.

This study, which attempted to clarify day-to-day fluctuations in behavioral performance in the natural daily life of athletes, was designed to eliminate the effects of physical factors and to isolate changes in nervous system factors as much as possible by adopting the CVM task. It is because this task requires no spatial bodily movement, which can decrease the influence of muscular factors such as muscle noise. However, the correlation coefficient between the MDD task score and CVM task score was not as high as expected, implying that these scores were influenced by uncontrollable factors such as physical factors followed by muscular contraction. Therefore, future research needs to consider how direct intervention in hMT+, such as rTMS and tDCS (transcranial direct current stimulation), affects the performance in both tasks.

### Visual motion perception and visuomotor response in athletes

Ball athletes have a superior visuomotor performance in response to visual motion compared to others, and the superiority is primarily related to visual perception (5, 6). The onset of visual motion, including coherent motion, evokes a potential with negativity N2 at around 150-200 ms at occipital and occipitotemporal electrode positions (13, 16). N2 has been suggested to reflect the perception/processing of visual motion information (5, 48, 49), and it has been reported that table tennis players and badminton players can react faster to the onset of visual motion and that their fast visuomotor response correlates with a short onset latency of N2 (5, 6). The onset latency of N2 has been reported to be reduced by an increase in visual motion speed (15), leading to an accelerated initiation of the motor response (14, 50). Similar results have been observed in the CVM task, where a faster target speed led to a shorter RCA start time [Supplementary Figure 1, (24)]. This finding was confirmed in the present study. Therefore, shortening the onset latency of N2, which reflects visual information processing in hMT+, shortens visual information processing of the visual motion signal, which in turn shortens the time until physical movement. This relationship may help explain the correlation between the performance in the MDD task and the RCA onset time in the CVM task observed in this study.

### Influence of table tennis experience on the functional connection between visual information processing and motor information processing

We found that only athletes with a history of table tennis for 8 years or more had a significant correlation between the discriminability of the direction of visual motion and the visuomotor ability (*r* = 0.64, *p* < 0.001). In particular, there was a moderate (*r* = −0.58, *p* < 0.001) correlation between the MDD task score and the RCA end-point position which is the spatial accuracy of the CVM task. In a real ball game, hitting a ball requires not only a quick reaction but also the spatial accuracy of visual and motor information processing. Therefore, we assumed the accumulation of repetitive experiences of visuomotor responses based on visual information strengthens the functional connection between visual information processing and motor information processing. Consistently, several reports have shown that motor skill learning through long-term training is associated with structural plasticity of the brain (51–56).

Using VBM (voxel-based morphometry), Draganski et al. (51) showed that training normal adults with no juggling experience increases gray matter near the middle temporal region of the visual cortex and the parietal lobe groove. Similarly, higher training intensity in golf was found to increase gray matter (57). However, the increase in the gray matter did not correlate with performance, so it may only reflect the amount of input; that is, the frequent use of specific neural circuits by the training. Therefore, strengthening the functional connection between visual information processing and motor information processing may reflect structural plastic changes in the brain more than short-term functional changes in neural circuits.

### Technical limitations

This study found a possibility that the day-to-day fluctuation of visuomotor response reflects that of visual motion discriminability. However, the cause of the fluctuations remains unknown. Various factors, such as arousal level, attentional function, physical and psychosocial stress, and emotional factors may affect visuomotor function (38, 39). Moreover, the factors of fluctuation may differ for each participant, therefore, in the future, it is necessary to investigate the relationship between visual information processing and visuomotor information processing using these physiological indicators. In addition, the effects of rTMS, tDCS, and tSMS (transcranial static magnetic field stimulation) on the direct relationship between visual motion discriminability and continuous visuomotor ability should be investigated (58). If the fluctuation of visuomotor ability reflects that of visual motion discriminability which is determined by the excitability of hMT+, it would be expected for both abilities to co-vary if the above interventions enhance or suppress hMT+ excitability.

## Conclusion

The daily performance of ball athletes varies due to their physical condition and sharpness of movements. This study shows that one of the reasons for the fluctuations in their visuomotor performance is the fluctuations in visual motion discriminability, the augmentation of which leads to improve performance.

## Data availability statement

The original contributions presented in the study are included in the article/supplementary material, further inquiries can be directed to the corresponding author/s.

## Ethics statement

The studies involving human participants were reviewed and approved by the Ethics Committee of the Graduate School of Medicine, Osaka University. Informed consent regarding the aim and experimental protocols of the present study was obtained in writing from all participants before participating in this study. The patients/participants provided their written informed consent to participate in this study.

## Author contributions

AT and SS conceived and designed the research, collected the data and performed the analyses, and wrote the main manuscript and prepared the figures and table. All authors interpreted the results of the experiments and read and approved the final version of the manuscript.

## Funding

This work was supported by a research grant from the Public Interest Incorporated Foundation Yamaha Motor Foundation for Sports and JST SPRING (Grant No. JPMJSP2138) to AT, KAKENHI (Grant Nos. JP20H04077 and JP19K22807 to SS and JP19K19977 to CA), and the Sports Research Innovation Project (SRIP) grant to SS.

## Conflict of interest

The authors declare that the research was conducted in the absence of any commercial or financial relationships that could be construed as a potential conflict of interest.

## Publisher's note

All claims expressed in this article are solely those of the authors and do not necessarily represent those of their affiliated organizations, or those of the publisher, the editors and the reviewers. Any product that may be evaluated in this article, or claim that may be made by its manufacturer, is not guaranteed or endorsed by the publisher.
